# Elevated expression of miR-142-3p is related to the pro-inflammatory function of monocyte-derived dendritic cells in SLE

**DOI:** 10.1186/s13075-016-1158-z

**Published:** 2016-11-16

**Authors:** Yilun Wang, Jun Liang, Haihong Qin, Yan Ge, Juan Du, Jinran Lin, Xiaohua Zhu, Jie Wang, Jinhua Xu

**Affiliations:** 1 Department of Dermatology, Huashan Hospital, Fudan University, 12 Wulumuqi Zhong Road, Shanghai, 200040 People’s Republic of China; 2 Department of Neurology, Huashan Hospital, Fudan University, Shanghai, People’s Republic of China; 3 Department of Human Anatomy and Histoembryology, School of Basic Medical Science, Fudan University, Shanghai, People’s Republic of China

**Keywords:** SLE, Monocyte-derived DCs, MicroRNA

## Abstract

**Background:**

Recent studies have shown that alterations in the function of dendritic cells (DCs) are involved in the pathogenesis of systemic lupus erythematosus (SLE). However, the mechanism of the alteration remains unclear.

**Methods:**

We cultured monocyte-derived DCs (moDCs) in vitro and examined the cytokines and chemokines in the supernatants of moDCs in negative controls (NC) and SLE patients in active phase. We then profiled microRNAs (miRNAs) of LPS-stimulated moDCs in SLE patients and used real-time PCR to verify the differentially expressed miRNAs. A lentiviral construct was used to overexpress the level of miR-142-3p in moDCs of NC. We examined the cytokines and chemokines in the supernatants of moDCs overexpressing miR-142-3p and used Transwell test, flow cytometric analysis and cell proliferation to observe the impact on CD4^+^ T cells in moDC-CD4^+^T cell co-culture.

**Results:**

moDCs in patients with SLE secreted increased level of IL-6, CCL2 and CCL5, with attraction of more CD4^+^ T cells compared with NC. We found 18 differentially expressed microRNAs in moDCs of SLE patients by microarray, and target gene prediction showed some target genes of differentially expressed miRNAs were involved in cytokine regulation. miR-142-3p was verified among the highly expressed miRNAs in the SLE group and overexpressing miR-142-3p in moDCs of the NC group caused an increase of SLE-related cytokines, such as CCL2, CCL5, CXCL8, IL-6 and TNF-α. Moreover, moDCs overexpressed with miR-142-3p resulted in attraction of an increased number of CD4^+^ T cells and in suppression of the proportion of Tregs in DC-CD4^+^T cell co-culture whereas the proliferation of CD4^+^T cells was not altered.

**Conclusions:**

The results demonstrated a role for miR-142-3p in regulating the pro-inflammatory function of moDCs in the pathogenesis of SLE. These findings suggested that miR-142-3p could serve as a novel therapeutic target for the treatment of SLE.

**Electronic supplementary material:**

The online version of this article (doi:10.1186/s13075-016-1158-z) contains supplementary material, which is available to authorized users.

## Background

Systemic lupus erythematosus (SLE) is a complicated autoimmune disease impairing multiple organs. The disease predominantly affects women aged 15-40 years [1] with a female to male ratio of 9:1 [2]. Both genetic and environmental factors contribute to human SLE pathogenesis [3], but the etiology of SLE is not fully understood. Impaired clearance of dying cells may represent a central pathogenic process in human lupus [4]. The accumulated dying cells release autoantigens, which are presented by dendritic cells, further breaking down the immune tolerance of T and B cells and triggering SLE.

Dendritic cells (DCs) are so far the most potent antigen-presenting cells (APCs) with important functions in the immune system. Immunodysregulation in SLE involves the complex interplay of various immune cells and DCs are the master regulators for initiation, amplification, and perpetuation of the disease [5]. DCs could influence SLE in several ways including: presentation of self-antigens to autoreactive T cells; oversecretion of pro-inflammatory cytokines; and suppression of regulatory T cells and promotion of B cell autoantibody production, either directly or indirectly [6–8]. However, the mechanisms of the altered function of DCs in SLE are largely unknown.

MicroRNAs (miRNAs) are small noncoding RNAs, which regulate protein expression at the posttranscriptional level through reduction of mRNA stability or inhibition of translation via binding to the 3′ un-translated regions (3′UTR) of target genes. They are involved in many kinds of diseases including tumors, infections, and autoimmune diseases. MicroRNAs can regulate DCs at different stages including differentiation, maturation, and apoptosis [9–12]. Monocyte-derived DCs (moDCs) from Blimp1 SLE-risk allele carriers exhibited increased expression of miRNA let-7c, which inhibited Blimp1 and also blocked lipopolysaccharide (LPS)-induced suppressor of cytokine signaling-1 (SOCS1) expression, contributing to the increased expression of pro-inflammatory cytokines [13]. The results suggested miRNAs in DCs might participate in the pathogenesis of SLE.

DC populations are relatively rare in blood. We therefore utilized the well-accepted model of human DC differentiation from peripheral blood monocytes under inflammatory conditions, which is a useful tool to study DC functions ex vivo [14–16]. Pathogenic inflammation can trigger SLE disease exacerbations [17, 18], in which Toll-like receptor 4 (TLR4) and TLR4 responsiveness are important [19]. We therefore choose LPS-activated moDCs as a subtype of DCs generated upon inflammation.

The aim of our study was to find any miRNAs participating in regulating the function of moDCs in SLE. In the study, we found moDCs in the SLE group produced increased levels of pro-inflammatory cytokines. Since all culture conditions were the same, the discrepancy between the SLE group and healthy control group was due to the intrinsic factors. MiRNA microarray analysis was conducted and the target genes of miRNAs significantly differentially expressed in patients with SLE were involved in cytokine regulation through target gene prediction. We further proved the expression level of miR-142-3p might influence the function of moDCs. Thus, we proposed a regulatory mechanism of miR-142-3p in moDCs of SLE.

## Methods

### Subjects

Fifteen female SLE patients whose SLE Disease Activity Index (SLEDAI) score was moderate to severe were recruited from the inpatient service in Huashan Hospital, Fudan University. Relevant clinical and laboratory information regarding the patients is shown in Table 1. The diagnostic criteria were in accordance with the 1997 American College of Rheumatology (ACR) revised criteria for the classification of SLE. Fifteen female healthy controls were also recruited. The study was approved by the Independent Ethics Committee of Huashan Hospital and written informed consent was obtained from all subjects. All the experiments were carried out in accordance with the relevant guidelines and regulations of Huashan Hospital.

**Table 1:** Clinical and laboratory characteristics of the patients with SLE in the study

Characteristics	SLE (n = 15)
Sex [male/female (n)]	0/15 (15)
Age (years) [median (range)]	33 (17–57)
SLEDAI score [median (range)]	15 (10–30)
Anti-dsDNA (IU/ml) [median (range)]	391.9 (25.9–800)
C3 (g/l) [median (range)]	0.6 (0.22–1.02)
C4 (g/l) [median (range)]	0.07 (0.06–0.15)
Red cell count (*10^12/l) [median (range)]	3.91 (3.16–4.44)
Lymphocyte count (*10^9/l) [median (range)]	4.74 (1.56–19.78)
Platelet count (*10^9/l) [median (range)]	181 (25–382)
Organ involvement [Y/N (n)]	6/9 (15)
Steroids [Y/N (n)]	9/6 (15)
Immunosuppressant [Y/N (n)]	0/15 (15)

*SLE* systemic lupus erythematosus, *SLEDAI* SLE Disease Activity Index, *dsDNA* double-stranded DNA

### Cell culture

EDTA blood (25 ml) was collected from each patient and control subject. Nearly 1–2*10^7 peripheral blood mononuclear cells (PBMCs) were isolated from each patient’s blood and CD14^+^ monocytes were sorted by positive selection (purity > 90 %) using magnetic beads (Miltenyi Biotec, Bergisch Gladbach, Germany). CD14^+^ monocytes were then cultured for 5–7 days in RPMI 1640 containing 10 % fetal bovine serum (FBS), penicillin/streptomycin solution and L-glutamine (Life Technologies, Carlsbad, CA, USA) supplemented with 1000 U/ml granulocyte-macrophage colony-stimulating factor (GM-CSF) and 1000 U/ml interleukin-4 (IL-4) every 2 days (PeproTech, Rocky Hill, NJ, USA). For transfection experiments, immature moDCs were harvested at day 5. For moDC maturation, 1 μg/ml LPS (*Escherichia coli* type 055:B6; Sigma-Aldrich, St. Louis, MO, USA) was added into the medium at day 6. The following antibodies were used to identify the phenotype of moDCs: anti-CD11c, anti-HLA-DR, anti-CD40, anti-CD86, anti-CD83 and low expression of anti-CD14 (eBioscience, San Diego, CA, USA).

### Lentiviral transfections

A fragment encoding miR-142-3p was amplified by PCR from human genomic DNA using the primers 5′-GCCACAAGGAGGGCTGGGGGGC-3′ and 5′-GAGCGCCGAGGAAGATGGTGGC-3′. Those fragments confirmed by sequencing were cloned into the pHBLV vector respectively (Hanbio, Shanghai, China). moDCs transfected with the empty lentiviral vector (designated as VEC) or RPMI 1640 medium (designated as NC) were used as controls. After 3 days, the transfected moDCs were collected.

### Microarray analysis and real-time PCR

Total RNA was extracted from moDCs using the miRNeasy Mini Kit (Qiagen, Hilden, Germany). miRNA expression profiling was determined by miRNA microarray analysis using the Agilent Human miRNA Array V19.0 ID:046064 (Agilent Technologies, Santa Clara, CA, USA) that included 2006 mature human miRNAs. Differentially expressed miRNAs were identified using the paired *t* test with the cutoff criteria of *P* < 0.05.

Reverse transcription was performed to obtain the cDNA for miRNA using the All-in-One miRNA qRT-PCR Detection Kit (Genecopoiea, Rockville, MD, USA). Quantitative real-time PCR was carried out with the Rotor-Gene Q (Qiagen) using the All-in-One miRNA qRT-PCR Detection Kit (Genecopoiea). The housekeeping gene U6 was used as the internal control. The primers for microRNAs and U6 were purchased from Genecopoiea directly.

### Target gene prediction

The target genes of differentially expressed miRNAs were predicted by at least two databases of the following five usual prediction databases: TargetScan (http://www.targetscan.org), miRanda (http://www.microrna.org/ microrna/home.do), PicTar (http://pictar.mdc-berlin.de/), MirTarget2 from miRDB (http://mirdb.org/miRDB/ download.html), and PITA (http://genie.weizmann.ac.il). Moreover, the Gene Ontology (GO) functional and pathway enrichment analysis were conducted for the target genes using the Database for Annotation, Visualization and Integrated Discovery (DAVID) online tools with the cutoff criterion of a false discovery rate (FDR) < 0.05.

### moDCs-CD4^+^ T cells co-culture

moDCs in each group were collected and overloaded with OVA peptide (Sigma-Aldrich) for 2 h, used as stimulator cells. They were suspended in RPMI 1640 medium to a final concentration of 5 × 10^5^/ml. Allogeneic CD4^+^ T cells were obtained from positive selection of PBMCs as responding cells. The density of responding CD4^+^ T cells was adjusted to 5 × 10^6^/ml. Stimulator cells and responding cells were added to each well on the 96-well plates. Each sample was tested in triplicate. The stimulator and responding cells were cultured together in an incubator (37 °C, 5 % CO_2_) for 3 days.

### CD4^+^CD25^+^Foxp3^+^ Tregs analysis

After co-culture for 3 days, cell suspensions were incubated with FITC-conjugated anti-human CD4 and PE-conjugated anti-human CD25 (Biolegend, San Diego, CA, USA) for 30 min at 4 °C and washed twice with 2 ml of phosphate-buffered saline (PBS) pH 7.4 containing 1 % bovine serum albumin (BSA). Intracellular staining for Foxp3 was then performed with APC-conjugated anti-human Foxp3 (eBioscience, San Diego, CA, USA) for 60 min and then washed with PBS/BSA. The supernatants were discarded and cells were resuspended in 0.2 ml PBS/BSA. Data were acquired with a FACSCanto system (Becton Dickinson, Franklin Lakes, NJ, USA) and analyzed using Flowjo software (Tree Star, Inc., Ashland, OR, USA). The expression levels of CD4, CD25 and Foxp3 were evaluated by calculating the percentage of cells expressing each protein.

### CD4^+^ T cells proliferation

CD4^+^ T cells were labeled with 5 μM carboxyfluorescein diacetate succinimidyl ester (CFSE, Molecular Probes, Eugene, OR, USA) first and then co-cultured with moDCs. After 3 days, CFSE dilution was analyzed using flow cytometric analysis. The proliferation experiment was evaluated using a division index in Flowjo software (Tree Star, Inc.).

### Chemotaxis assay

CD4^+^ T cells were placed on the upper chamber of a Transwell plate, 6.5 mm in diameter, with 5-μm polycarbonate filters (Corning, Corning, NY, USA). The lower chamber contained either diluted moDC supernatant (1:1 with medium) or control medium. After culture of 3 h at 37 °C, the cells that had migrated to the lower chamber were harvested and counted under a light microscope.

### Chemokine and cytokine assays

Chemokines [C-X-C motif ligand (CXCL)8, C-C motif ligand (CCL)2 and CCL5] and cytokines [IL-6, tumor necrosis factor alpha (TNF-α), IL-10 and IL-17] in supernatants of moDCs or supernatants of CD4^+^ T cells-moDCs co-culture were simultaneously quantified using the Cytometric Bead Array (CBA) reagent kits (BD Biosciences Pharmingen, San Diego, CA, USA).

### Statistics

Continuous variables were expressed as mean (SD) and categorical variables as frequencies (%). The Student *t* test or one-way analysis of variance was used to compare continuous variables. All *P* values were estimated in a two-tailed fashion. Differences were considered to be statistically significant at *P* < 0.05. Data were analyzed using SPSS 13.0 (SPSS Inc., Chicago, IL, USA).

## Results

### Pro-inflammatory function of moDCs in SLE

**Fig. 1:**
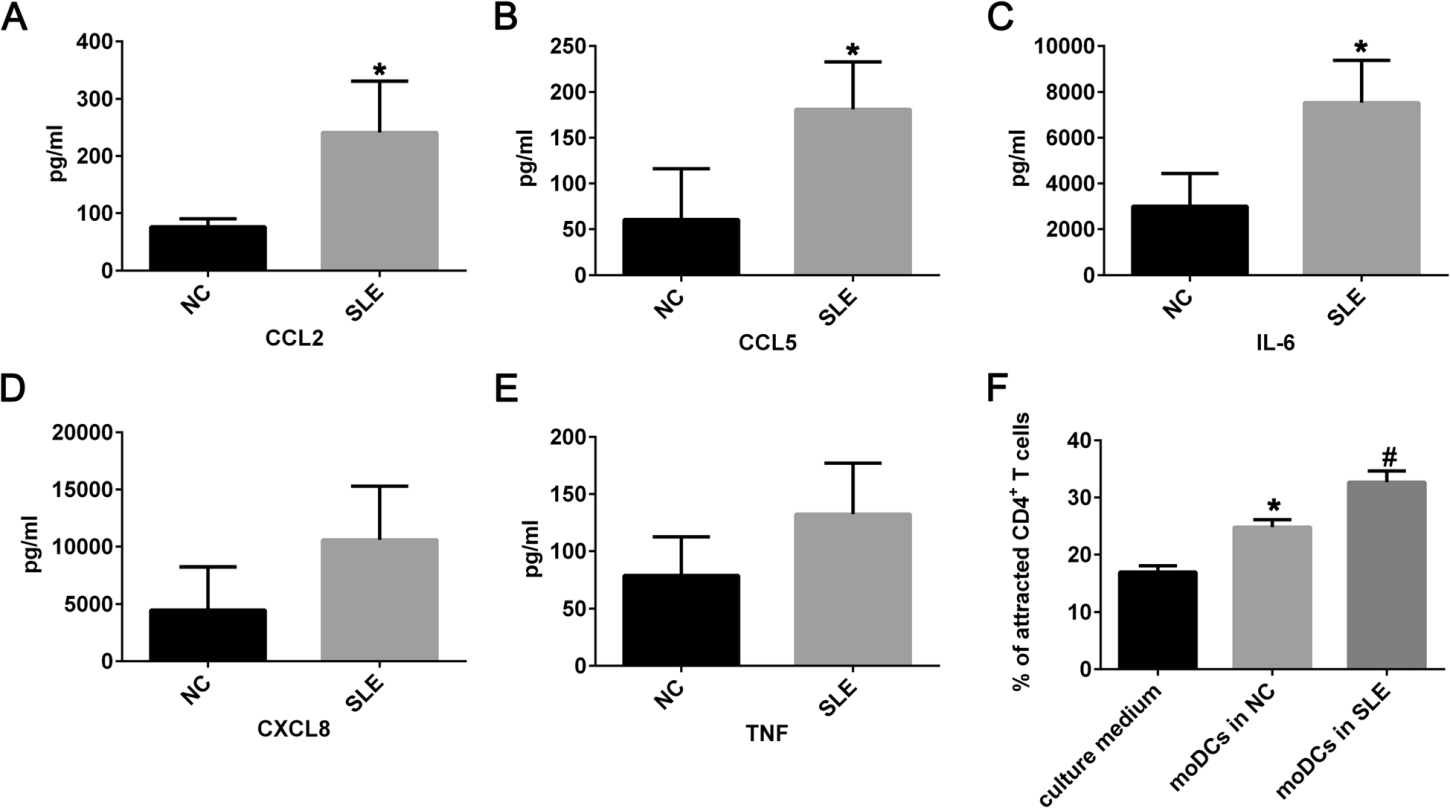
Pro-inflammatory function of moDCs in SLE. The supernatants derived from culture medium of moDCs from negative controls (n = 5) and patients with SLE (n = 5) in the presence of LPS (1 μg/ml) for 24 hours was assessed using cytometric bead array including CCL2 (**a**, *P < 0.001), CCL5 (**b**, *P = 0.002), IL-6 (**c**, *P = 0.002), CXCL8 (**d**, no significant difference) and TNF-α (**e**, no significant difference). Percentage of CD4^+^ T cells attracted by supernatants of culture medium, supernatants of moDCs in NC, and supernatants of moDCs in SLE group (**f**, ^#^ *P* < 0.05 versus supernatants of culture medium and supernatants of moDCs in NC, *P < 0.05 versus supernatants of culture medium). Data were shown as mean ± SD. *CCL* C-C motif ligand, *CXCL* C-X-C motif ligand, *IL* interleukin, *moDCs* monocyte-derived DCs, *NC* negative controls, *SLE* systemic lupus erythematosus, *TNF-*α tumor necrosis factor alpha

Since cytokine production was one of the major functions of DCs with great biological importance, we first investigated whether the secreted concentration of some cytokines and chemokines in the supernatants of moDCs was different between SLE patients and healthy controls. The result showed mature moDCs from patients with SLE produced significantly higher levels of IL-6, CCL2 and CCL5 compared with mature moDCs from healthy controls (Fig. 1a–c). We also measured levels of TNF-α and CXCL8 in cell culture supernatants between the two groups without finding significant differences (Fig. 1d and e). In addition, infiltration of T lymphocytes and other leukocytes into the sites of inflammation is important in SLE. We then found that supernatants of moDCs in the SLE group attracted significantly more allogeneic CD4^+^ T cells than the control group and culture medium group though a Transwell assay (Fig. 1f).

### miRNA profiling in LPS-activated moDCs of patients with SLE

**Fig. 2:**
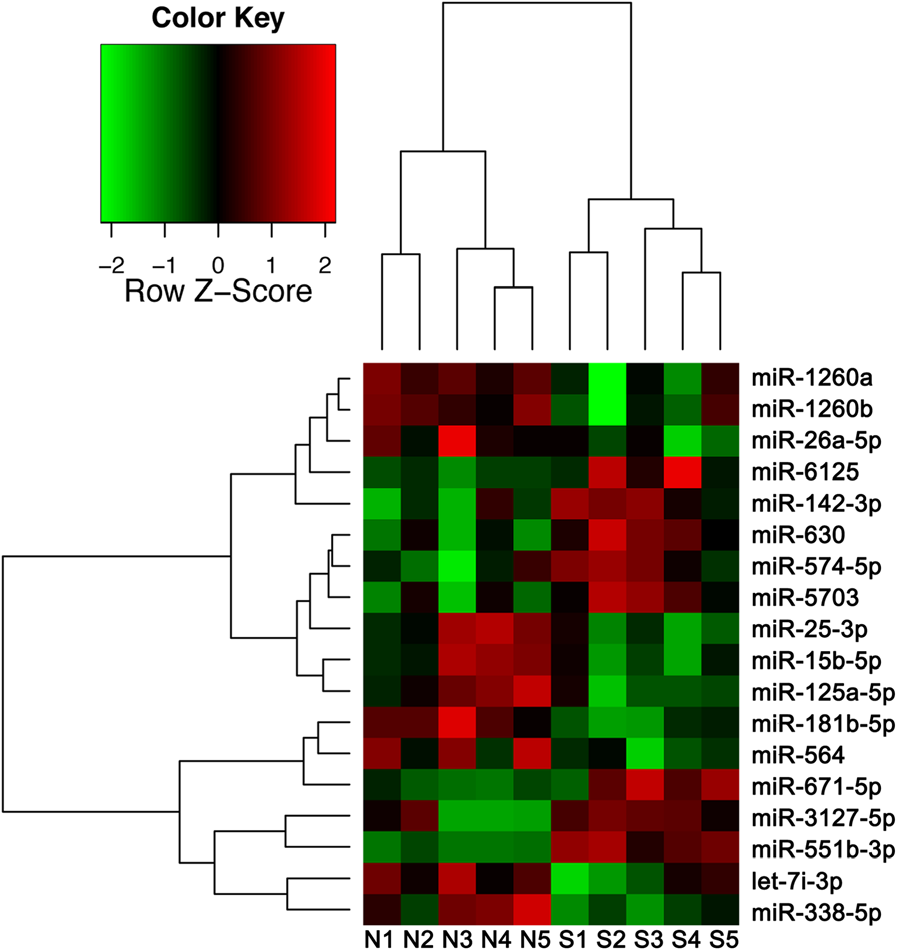
miRNA profiling in LPS-activated moDCs of patients with SLE. The expression levels of mature miRNAs purified from 24-h LPS-activated moDCs were analyzed using miRNA microarrays and hierarchical clustering of statistically significant differential miRNAs with analysis of variance (*P* < 0.05). N = 10. N1-N5 represented negative controls. S1-S5 represented the patients with SLE

miRNAs were able to regulate the function of DCs and we therefore investigated whether these noncoding RNAs would impact the function of moDCs in patients with SLE. We first performed miRNA microarrays to identify the expression levels of matured miRNAs purified from 24-h LPS-activated moDCs from five SLE patients who were in the active phase of the disease and five negative controls. Microarrays identified 18 miRNAs that were differentially regulated in SLE (Fig. 2). Table 2 showed the fold change and *P* value of the differentially regulated miRNAs. In all, the expression of eight miRNAs (miR-142-3p, miR-551b-3p, miR-3127-5p, miR-671-5p, miR-630, miR-5703, miR-6125, and miR-574-5p) was significantly increased in the patients with SLE, whereas the expression of ten miRNAs (miR-338-5p, let-7i-3p, miR-181b-5p, miR-1260b, miR-125a-5p, miR-1260a, miR-15b-5p, miR-25-3p, miR-26a-5p, and miR-564) was significantly decreased.

**Table 2:** Fold change and *P* values of differentially expressed miRNAs with *P* < 0.05

Expression miRNA in patients of SLE	Fold change	*P* value
Increased		
hsa-miR-551b-3p	55.1	<0.001
hsa-miR-3127-5p	3.3	0.044
hsa-miR-671-5p	2.7	0.024
hsa-miR-630	2.2	0.010
hsa-miR-5703	2.1	0.023
hsa-miR-6125	1.5	0.047
hsa-miR-142-3p	1.5	0.021
hsa-miR-574-5p	1.4	0.048
Decreased		
hsa-miR-338-5p	0.1	0.014
hsa-let-7i-3p	0.2	0.036
hsa-miR-181b-5p	0.4	0.003
hsa-miR-1260b	0.6	0.042
hsa-miR-125a-5p	0.6	0.017
hsa-miR-1260a	0.6	0.042
hsa-miR-15b-5p	0.7	0.028
hsa-miR-25-3p	0.8	0.020
hsa-miR-26a-5p	0.8	0.042
hsa-miR-564	0.9	0.030

*miRNA* microRNA, *SLE* systemic lupus erythematosus

Next, we performed functional and pathway enrichment analysis for target genes of all the differentially expressed miRNAs. The top ten functional and pathway enrichment analysis results are indicated in Additional files 1 and 2 respectively.

### Validation of differentially expressed miRNAs in the SLE group

**Fig. 3:**
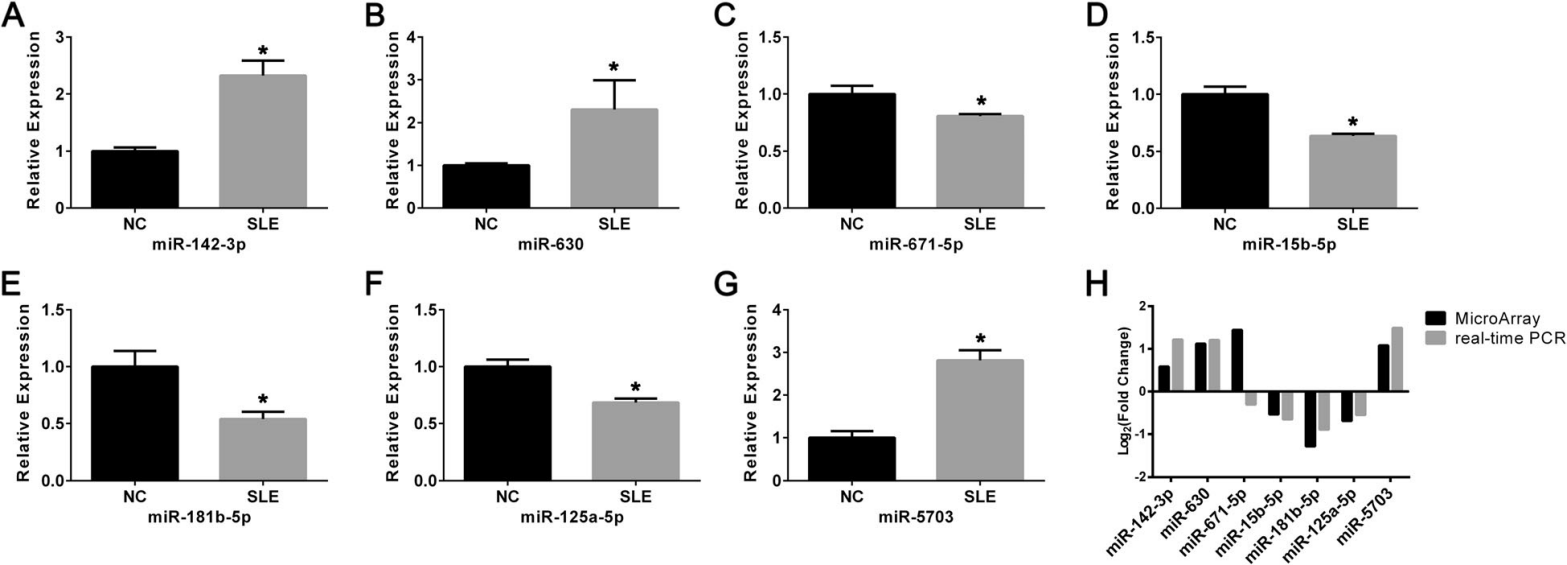
Validation of selected miRNAs by qRT-PCR. The levels of miR-142-3p (*P < 0.001), miR-630 (*P = 0.072), miR-671-5p (*P = 0.021), miR-15b-5p (*P = 0.005), miR-181b-5p (*P < 0.001), miR-125a-5p (*P < 0.001), and miR-5703 (*P < 0.001) were determined in LPS-activated moDCs of 15 negative controls (NCs) and 15 patients with SLE (**a**–**g**). Data were shown as mean ± SD. The change patterns between microarray analysis and qRT-PCR were shown in (**h**). *NC* negative controls, *PCR* polymerase chain reaction, *SLE* systemic lupus erythematosus

Seven differentially expressed miRNAs (miR-142-3p, miR-630, miR-671-5p, miR-15b-5p, miR-181b-5p, miR-125a-5p, and miR-5703) whose target genes were significantly enriched in important functions and pathways were selected for further validation. We used quantitative real-time PCR (qRT-PCR) to validate the microarray findings among 15 patients and 15 healthy controls including those five patients and five healthy controls used in the microarray analysis (Fig. 3). The result of qRT-PCR revealed that six miRNAs (miR-142-3p, miR-630, miR-15b-5p, miR-181b-5p, miR-125a-5p, and miR-5703) showed the same change patterns as shown in the microarray analysis. However, the expression level of miR-671-5p was significantly decreased to 0.81-fold of NC (*P* = 0.021), which was opposite to the microarray analysis (Fig. 3h). This discrepancy might be due to technical limitations of the microarray, such as cross-hybridization, signal saturation, and limited dynamic range.

Among all the differentially expressed miRNAs, miR-142-3p attracted our attention. miR-142-3p was demonstrated to be essential for the specification of the hemangioblastic precursors of the blood stem cell lineage and might facilitate hematopoietic stem cells formation [20]. It was reported that miR-142^-/-^ mice developed abnormal hematopoietic lineages [21], and displayed an impairment of CD4^+^ DC homeostasis both in vitro and in vivo, leading to a severe and specific defect in the priming of CD4^+^ T cells [22]. On the other hand, the enhanced expression of miR-142-3p reduced the immunosuppressive activity of bone marrow myeloid-derived suppressor cells, restored CD8^+^ T cell proliferation and was able to change the ratio of macrophage: DC to favor the DC expansion [23]. In our study, we found miR-142-3p in moDCs was upregulated 2.33-fold in SLE patients (*P* < 0.001) (Fig. 3a), and moDCs of SLE patients secreted increased pro-inflammatory cytokines together with attracting more CD4^+^ T cells. Our functional enrichment analysis also indicated that target genes of miR-142-3p were involved in cytokine and chemokine regulation. As a result, we hypothesized that overexpression of miR-142-3p in moDCs might impact the function of moDCs and then lead to overactive immune reaction in the pathogenesis of SLE. We thus selected miR-142-3p for further study.

### Overexpression of miR-142-3p promoted pro-inflammatory function of moDCs

**Fig. 4:**
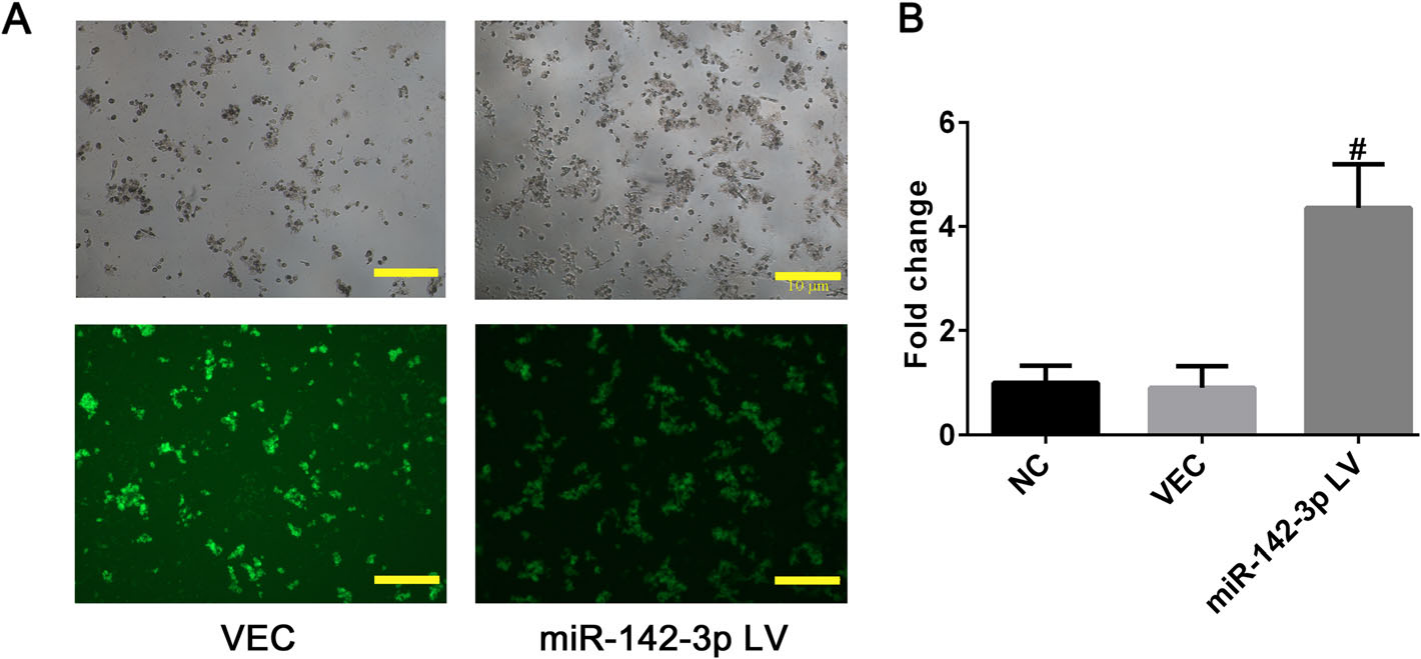
Transfection efficiency of overexpressing miR-142-3p in moDCs. **a** Morphology and GFP fluorescence of moDCs transfected with empty lentivirus vector (VEC) and miR-142-3p overexpressing lentivirus (LV). Scale bar = 10 μm. **b** The level of miR-142-3p expressed in moDCs of negative controls (NC), VEC or LV group was assessed by quantitative real-time PCR. Data were expressed as mean ± SD. Each experiment was conducted at least three times. ^#^ *P* < 0.005 versus NC and VEC

We investigated the potential role of miR-142-3p in moDCs through regulating the basal expression level of miR-142-3p. Transfection efficiency was determined by observing the fluorescent cells (Fig. 4a) and expression level of miR-142-3p in moDCs by qRT-PCR. The expression of miR-142-3p increased 4.79-fold in the miR-142-3p lentivirus (LV) group compared with the empty lentiviral vector (VEC) group while no significance was found between the VEC and NC group (Fig. 4b).

**Fig. 5:**
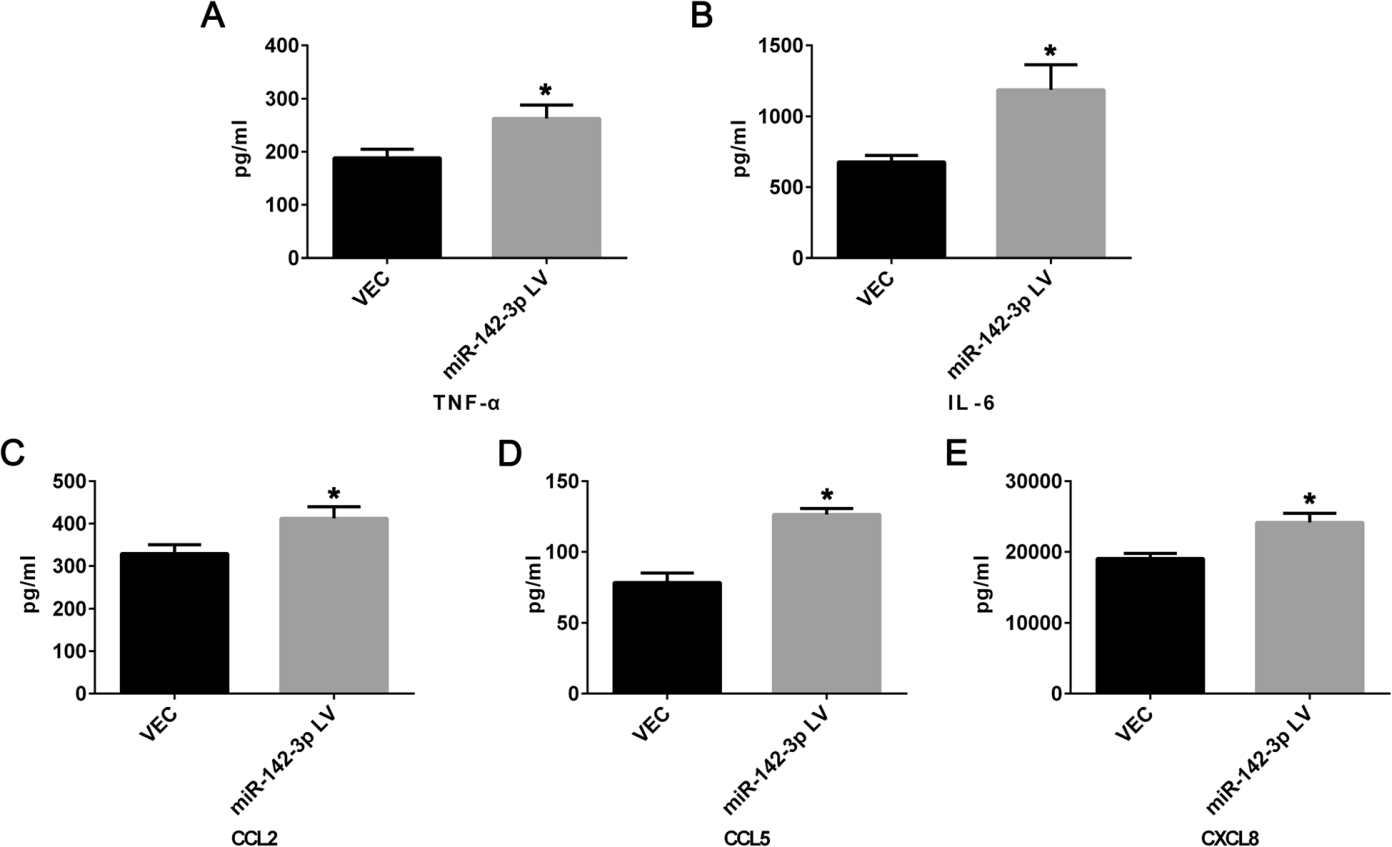
Overexpression of miR-142-3p promoted pro-inflammatory function of moDCs. The level of exocrine secretion of TNF-α (**a**, *P = 0.013), IL-6 (**b**, *P = 0.03), CCL2 (**c**, *P = 0.014), CCL5 (**d**, *P < 0.001) and CXCL8 (**e**, *P = 0.004) in the supernatant derived from moDCs in miR-142-3p lentivirus (LV) group compared with empty lentivirus vector (VEC). N = 3 in each group. Data were shown as means ± SD. *CCL* C-C motif ligand, *CXCL* C-X-C motif ligand, *IL* interleukin, *TNF-*α tumor necrosis factor alpha

Then, we investigated whether the expression level of miR-142-3p impacted the production of cytokines and chemokines secreted by moDCs since these soluble factors were expected to modulate CD4^+^ T cells-moDCs interaction. We examined the supernatants of moDCs in the VEC and LV group. We found the level of exocrine secretion of IL-6 (1187.65 ± 174.91 pg/ml, *P* = 0.03) and TNF-α (262.99 ± 25.33 pg/ml, *P* = 0.013) were significantly increased in the supernatants of moDC-overexpressed miR-142-3p compared with the VEC group (Fig. 5a and b). As shown in Fig. 5c–e, the level of exocrine secretion of CXCL8 (24171.17 ± 1294.77 pg/ml, *P* = 0.004), CCL2 (412.20 ± 27.61 pg/ml, *P* = 0.014) and CCL5 (126.46 ± 4.25 pg/ml, *P* < 0.001) was also significantly increased in LV group compared with VEC group.

### Elevated expression level of miR-142-3p affected moDCs-CD4^+^ T cells interaction

**Fig. 6:**
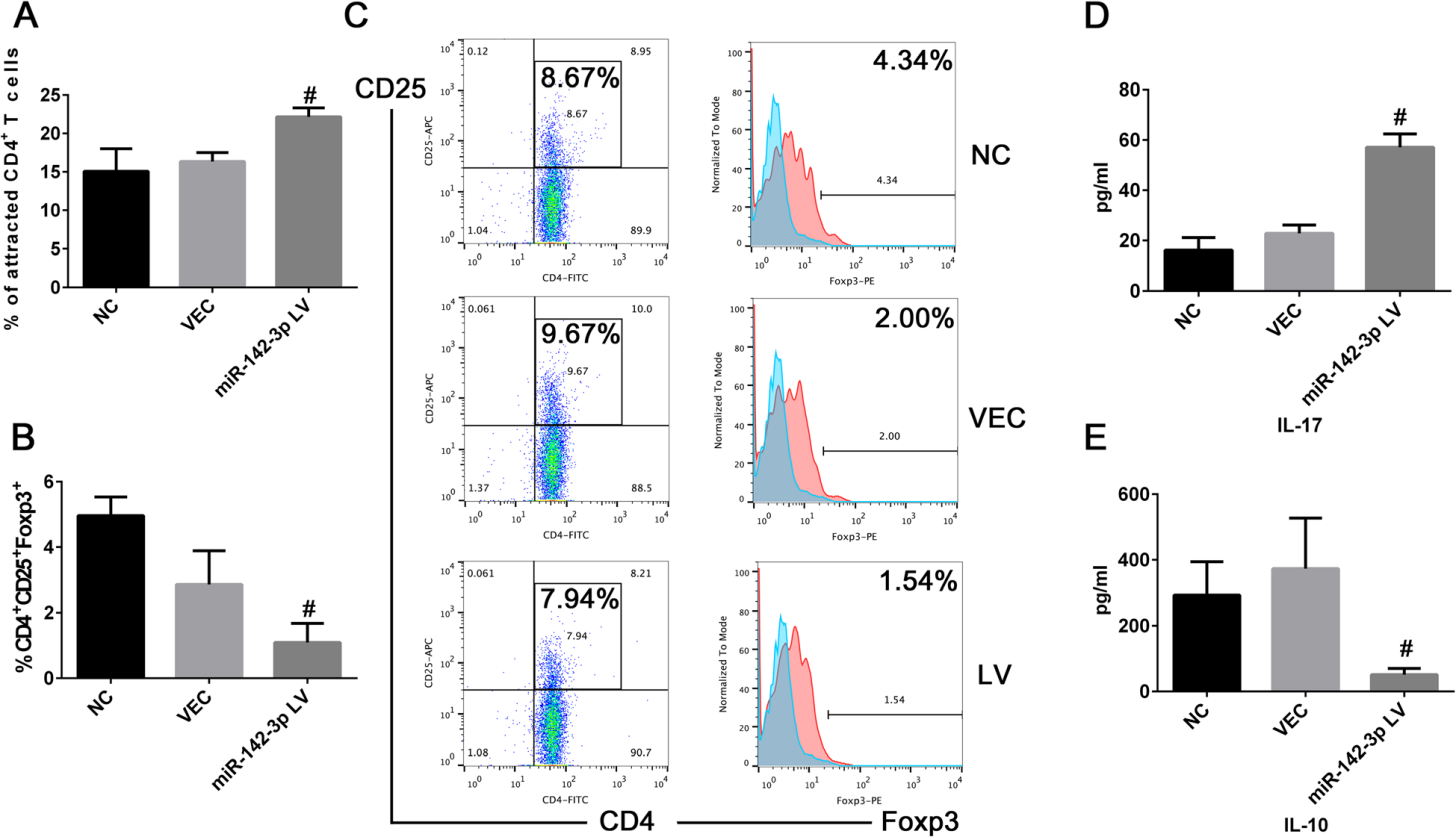
Elevated expression level of miR-142-3p affected moDCs-CD4^+^ T cells interaction. **a** Percentage of CD4^+^ T cells attracted by supernatants of moDCs in NC or empty lentiviral vector (VEC) or lentivirus (LV) group measured by a Transwell chamber. ^#^ *P* < 0.05 versus NC and VEC. **b** The percentage of CD4^+^CD25^+^Foxp3^+^ Tregs among CD4^+^ T cells co-cultured with moDCs was assessed using flow cytometry and data of one representative experiment (**c**) out of three independent experiments was shown. Foxp3^+^ cells were gated from CD4^+^CD25^+^ T cells. Data were shown as means ± SD of three independent experiments. ^#^ *P* < 0.05 versus NC and VEC. **d** and **e** The level of exocrine secretion of IL-17 (^#^ *P* < 0.001 versus NC and VEC) and IL-10 (^#^ *P* < 0.05 versus NC and VEC) in the supernatants derived from co-culture medium of CD4^+^T cells- moDCs. N = 3 in each group. Data were shown as means ± SD. *IL* interleukin

DC-induced T cell priming results in a robust immune response and is vital in SLE pathogenesis [24]. To assess a possible effect of miR-142-3p on the function of moDCs reacting on CD4^+^ T cells, we first used a Transwell assay to characterize the allogeneic CD4^+^ T cells attracted by supernatant of moDCs. As presented in Fig. 6a, supernatants in the miR-142-3p overexpression group attracted significantly more CD4 T^+^ cell migration to the lower chamber compared with the NC and VEC group. Next, moDCs were pulsed with ovalbumin (OVA) for 2 hours and were subsequently co-cultured with allogeneic CD4^+^ T cells. As a functional test, we compared CD4^+^ T cells stimulatory capacities between moDCs transfected with miR-142-3p overexpression lentivirus, or with empty vector, or with RPMI 1640 in an allogeneic moDCs-CD4^+^ T cells co-culture. The division index of CD4^+^ T cells in each group was 4.60 ± 0.19 (NC), 4.52 ± 0.31 (VEC), 4.73 ± 0.06 (LV) respectively. No significant differences were found among the ability of moDCs in NC, VEC or LV group to activate CD4^+^ T cells proliferation, indicating that miR-142-3p might not be able to enhance CD4^+^ T cells proliferation. However, the proportion of CD4^+^CD25^+^Foxp3^+^ Tregs among CD4^+^ T cells was significantly lower when CD4^+^ T cells were co-cultured with moDCs of LV group (1.09 ± 0.59 %) compared with NC (4.96 ± 0.57 %, *P* = 0.001) or VEC group (2.86 ± 1.03 %, *P* = 0.029) (Fig. 6b and c). We further examined the supernatants of co-culture medium and found the level of IL-17 was significantly higher in the LV group and the level of IL-10 was significantly decreased (Fig. 6d and e).

## Discussion

SLE is an autoimmune disease characterized by autoreactive B and T cells [25]. There is growing evidence indicating that alterations in the function of DCs, which are APCs capable of inducing activation of naive T cells, is related to the pathogenesis of SLE [25–27]. Given the challenges of tissue acquisition in humans, studies of DC subsets are carried out typically on DC generated in vitro from precursors. As a result, we use monocytes-derived DCs, which is a classic method to obtain enough DCs in vitro and has been successfully used as DCs according to its morphology, phenotype and function [15, 28, 29].

In the current work, we found moDCs in patients with SLE in their active phase secreted higher level of IL-6, CCL2, and CCL5 as well as attracting more CD4^+^ T cells. We then performed microarray and found miR-142-3p was highly expressed in human moDCs among the aberrant miRNAs in the SLE group. The function of moDCs was affected by altering the expression of miR-142-3p. We further found that the pro-inflammatory cytokine IL-6, TNF-α and chemokine CCL2, CCL5, CXCL8 were elevated in the miR-142-3p lentivirus group. Moreover, enforced miR-142-3p in moDCs could affect moDC-CD4^+^ T cell interaction through inhibiting the proliferation of CD4^+^CD25^+^Foxp3^+^ Tregs and attracting more CD4^+^ T cells. Thus, we concluded miR-142-3p promoted pro-inflammatory function of moDCs and possibly contributing to the pathogenesis of SLE.

It is known from animal lupus models and from studies in patients with lupus nephritis that inflammatory chemokines, especially CCL2 and CCL5, were readily detectable in the kidney tissue and urine [30, 31]. IL-6 is significantly elevated in the body fluids of SLE patients, including serum, plasma, and cerebrospinal fluid [32–35]. Therefore, the pathogenesis of SLE involves altered cytokine production. Similarly, we have found moDCs of patients with SLE in active phase could produce higher levels of IL-6, CCL2, and CLL5, which might contribute to explain the dynamic role of these cells in disease pathogenesis. Since moDCs in our study were differentiated under the same conditions, the discrepancy between the SLE group and healthy control group was due to the intrinsic factors. It was reported monocytes in patients with SLE produced higher IL-6 and TNF-α [36], and presented an increase of CCL2, CXCL9, and CXCL10 mRNA expression when compared with control group [37]. Therefore, both monocyte-derived DCs and their parental monocytes in SLE displayed pro-inflammatory function.

miR-142 is exclusively expressed in hematopoietic cells under homeostatic conditions and has been shown to play a critical role in immune responses, such as regulating HMGB1 in THP-1 cells [38], SOCS1 in human macrophages [39], and cAMP in mice Treg cells [40]. We found miR-142-3p increased in monocyte-derived DCs from patients with SLE, which is consistent with the upregulation of miR-142-3p in B cells [41], PBMCs [42], and plasma [43] from SLE patients. In our study, functional enrichment analysis indicated that target genes of miR-142-3p (IL6ST, SPRED1, BCL2L1, STAM, IL7R, LIFR, PRLR) and (ITGB8, ITGAV, CRK, ROCK2, COL24A1, RAC1) were significantly enriched in the Jak-STAT signaling pathway and focal adhesion pathway respectively, which were important pathways for cytokines and chemokines. It has been reported these two pathways are both associated with SLE [44, 45]. Therefore, it could be speculated that miR-142-3p might play important roles in SLE by regulating their target genes that participate in these important signaling pathways.

Leukocyte migration is mediated by the interaction of a number of chemokines and their receptors [46]. These small molecules have well-defined roles in directing cell movements necessary for the initiation of T cell immune response, attraction of appropriate effector cells to sites of inflammation, and regulation of differential recruitment of T helper lymphocytes. Matured DCs could secrete an abundant source of both inflammatory and lymphoid chemokines, sustaining interaction of naive and activated T cells with antigen-presenting mature DCs. In our experiments, we found the level of CXCL8, CCL2, and CCL5 increased in the supernatants of moDCs with enforced expression of miR-142-3p. The three chemokines have also been reported in biological fluids from SLE [31, 47], so they might act synergistically on the chemotactic activity inducing CD4^+^ T cell migration. Since miR-142-3p is elevated in moDCs from SLE patients, it suggests that the upregulation of miR-142-3p in moDCs of SLE patients might be attributed to attracting more CD4^+^ T cells, probably involved in the pathogenesis of disease.

We also found the overexpression of miR-142-3p cause the elevation of IL-6 and TNF-α, leading to a decrease of CD4^+^CD25^+^Foxp3^+^ Tregs which are anti-inflammatory, and an imbalance of IL-17 and IL-10. Treg deficiency in the periphery is sufficient to evoke chronic T cell-mediated autoimmunity and immunopathology, which has been associated with SLE [48]. The mechanisms of Treg-mediated suppression including secretion of immunosuppressive cytokines, cell contact-dependent suppression, and functional modification or killing of APC [48]. It has been demonstrated in lupus-prone mice that IL-6 produced by DCs inhibits Tregs [49], because Foxp3^+^ Treg cells lose Foxp3 expression and undergo conversion into Th17 cells under the effect of IL-6 [50]. Therefore, IL-6 triggered an immune disorder by breaking the balance between Th17 and Treg. Our results indicate that the increase of miR-142-3p in moDCs of SLE made the cells producing increased IL-6 and could induce CD4^+^ T cells to secrete more IL-17 and less IL-10, thus leading to an imbalance of the immune response. Therefore, overexpression of miR-142-3p in moDCs suppressed the increase in Tregs, which correlated with a reduced capacity to suppress responder T cell proliferation and might thereby contribute to the development of SLE. Similarly, mouse dendritic cells matured by ingestion of apoptotic blebs could stimulate allogeneic T cells which produced IFN and especially high levels of IL-17, representing an important driving force in SLE [51].

We have suggested that elevated expression of miR-142-3p is related to the pro-inflammatory function of moDCs in SLE. However, the limitation of our study is that we have not investigated the effect of decreasing the level of miR-142-3p in moDCs of SLE. Future study would focus on the exact target genes of miR-142-3p in moDCs and whether downregulating miR-142-3p could improve the overactive inflammation phase of SLE.

## Conclusion

Taken together, our findings suggested a pro-inflammatory function of moDCs in SLE patients partially mediated by miR-142-3p: (1) microRNAs were differentially expressed and miR-142-3p was increased in moDCs of SLE patients. (2) moDCs in patients with SLE produced higher levels of some pro-inflammatory cytokines and chemokines than healthy controls. (3) Overexpressing miR-142-3p in moDCs of healthy controls promoted cytokine and chemokine production to attract more CD4^+^ T cells and decrease Treg expansion. These findings suggested that miR-142-3p might be meaningful in the pathogenesis of SLE and could serve as a novel therapeutic target for treatment.

## Additional files

Additional file 1: Table S1. The top ten enriched Gene Ontology (GO) terms for target genes of all the 18 differentially expressed miRNAs. (DOCX 72 kb)
Additional file 2: Table S2. The top ten enriched pathways for target genes of all the 18 differentially expressed miRNAs. (DOCX 68 kb)

## Abbreviations

3′UTR: 3′ un-translated regions
APC: Antigen-presenting cells
BSA: Bovine serum albumin
CCL: C-C motif ligand
CFSE: Carboxyfluorescein diacetate succinimidyl ester
CXCL: C-X-C motif ligand
DCs: Dendritic cells
FBS: Fetal bovine serum
FDR: False discovery rate
GM-CSF: Granulocyte-macrophage colony-stimulating factor
IL: Interleukin
LPS: Lipopolysaccharide
LV: Lentivirus
miRNAs: microRNAs
moDCs: monocyte-derived DCs
NC: Negative controls
OVA: Ovalbumin
PBMCs: Peripheral blood mononuclear cells
PBS: Phosphate-buffered saline
qRT-PCR: quantitative real-time PCR
SLE: Systemic lupus erythematosus
SOCS1: Suppressor of cytokine signaling-1
TLR: Toll-like receptor
TNF-α: Tumor necrosis factor alpha
Tregs: Regulatory T cells
VEC: Empty lentiviral vector
