# Supplementary material for: Elevated expression of miR-142-3p is related to the pro-inflammatory function of monocyte-derived dendritic cells in SLE
Source: Arthritis Res Ther. 2016 Nov 16;18:263. doi: 10.1186/s13075-016-1158-z (PMC5112667; doi:10.1186/s13075-016-1158-z)
Supplement: Additional file 1: Table S1. — The top ten enriched Gene Ontology (GO) terms for target genes of all the 18 differentially expressed miRNAs. (DOCX 72 kb) [file 13075_2016_1158_MOESM1_ESM.docx]

**Table S1. The top ten enriched Gene Ontology (GO) terms for target genes of all the 18 differentially expressed miRNAs**

| GO ID | GO term | TYPE | TargetGene in GO | TargetGene list | FDR |
| --- | --- | --- | --- | --- | --- |
| GO:0007156 | homophilic cell adhesion | biological_process | 45 | CDH2,PCDHGC3,PCDH9,RET,ROBO2,etc. | <0.001 |
| GO:0008270 | zinc ion binding | molecular_function | 225 | XIAP,ZFHX3,BCL6,ZFP36L1,ZFP36L2,etc. | <0.001 |
| GO:0005515 | protein binding | molecular_function | 477 | ABL1,ACVR2A,ACVR2B,ADM,ADRB2,etc. | <0.001 |
| GO:0004930 | G-protein coupled receptor activity | molecular_function | 12 | BAI3,CASR,S1PR1,LPAR1,GPR4,etc. | <0.001 |
| GO:0006351 | transcription, DNA-dependent | biological_process | 182 | BCL6,RUNX1T1,CCNT2,CHD4,KLF6,etc. | 0.003 |
| GO:0005634 | nucleus | cellular_component | 423 | ABL1,ADARB1,ADCY1,ADRB2,XIAP,etc. | 0.012 |
| GO:0003700 | sequence-specific DNA binding transcription factor activity | molecular_function | 100 | ZFHX3,BCL6,ZFP36L1,ZFP36L2,RUNX2,etc. | 0.013 |
| GO:0045944 | positive regulation of transcription from RNA polymerase II promoter | biological_process | 80 | ADRB2,ARNTL,RUNX2,CCNT2,CD28,etc. | 0.012 |
| GO:0010863 | positive regulation of phospholipase C activity | biological_process | 5 | FGF2,FGFR1,FLT1,KIT,PDGFRA | 0.048 |
| GO:0035098 | ESC/E(Z) complex | cellular_component | 6 | EZH1,EZH2,RBBP7,EED,SIRT1,etc. | 0.047 |

FDR: false discovery rate.
