# Supplementary material for: Elevated expression of miR-142-3p is related to the pro-inflammatory function of monocyte-derived dendritic cells in SLE
Source: Arthritis Res Ther. 2016 Nov 16;18:263. doi: 10.1186/s13075-016-1158-z (PMC5112667; doi:10.1186/s13075-016-1158-z)
Supplement: Additional file 2: Table S2. — The top ten enriched pathways for target genes of all the 18 differentially expressed miRNAs. (DOCX 68 kb) [file 13075_2016_1158_MOESM2_ESM.docx]

**Table S2. The top ten enriched pathways for target genes of all the 18 differentially expressed miRNAs.**

| pathway ID | KEGG name | TragetGene in this pathway | Gene list | FDR |
| --- | --- | --- | --- | --- |
| hsa04740 | Olfactory transduction | 2 | CALM1,PDE1C, | <0.001 |
| hsa04510 | Focal adhesion | 29 | MYLK3,ITGB8,ITGA4,ITGAV,FLT1,etc. | 0.016 |
| hsa04151 | PI3K-Akt signaling pathway | 40 | CCNE1,RPS6KB1,HSP90B1,ITGB8,GHR,etc. | 0.051 |
| hsa05215 | Prostate cancer | 15 | CCNE1,EP300,HSP90B1,SRD5A2,CCNE2,etc. | 0.039 |
| hsa05211 | Renal cell carcinoma | 12 | EP300,PAK7,RAP1B,PIK3R3,MAPK1,etc. | 0.047 |
| hsa04810 | Regulation of actin cytoskeleton | 27 | MYLK3,ITGB8,ITGA4,ITGAV,BAIAP2,etc. | 0.050 |
| hsa04660 | T cell receptor signaling pathway | 16 | MAP2K7,PAK7,PIK3R3,MAPK1,CD28,etc. | 0.043 |
| hsa04710 | Circadian rhythm | 7 | RORB,ARNTL,CLOCK,BHLHE40,BTRC,etc. | 0.052 |
| hsa04015 | Rap1 signaling pathway | 26 | ADCY1,CNR1,FLT1,CALM1,RAP1B,etc. | 0.059 |
| hsa04666 | Fc gamma R-mediated phagocytosis | 14 | PPAP2A,RPS6KB1,PIK3R3,WASL,MAPK1,etc. | 0.055 |

FDR: false discovery rate
